# The younger frail critically ill patient: a newly recognised phenomenon in intensive care?

**DOI:** 10.1186/s13054-016-1526-8

**Published:** 2016-11-01

**Authors:** Stephen Bonner, Nazir I. Lone

**Affiliations:** 1 James Cook University Hospital, Middlesbrough, UK; 2 Usher Institute of Population Health Sciences and Informatics, University of Edinburgh, Edinburgh, UK

Clinicians have used the term ‘frailty’ in everyday practice for many decades and have felt able to identify such individuals despite the lack of agreed clinical definitions. However, advances in conceptualising and defining frailty through more objective criteria have emerged, allowing frailty to be investigated in wider settings [1]. Frailty is described as a clinical picture of loss of physiological and cognitive functioning which leaves patients susceptible to significant deterioration often precipitated by relatively minor stressors, such as infection, surgery or trauma [2]. The syndrome described by Fried et al. [2] has three or more characteristics, such as unintentional weight loss, exhaustion, weakness, slow walking speed and low physical activity. This ‘frailty phenotype’ results in falls, frequent hospitalisation and death.

Whilst this syndrome is usually described in the elderly, it is increasingly recognised that it is also present in younger age groups. Interest is increasing in the management of this population in many fields of medicine, including critical care and perioperative medicine, where risk stratification of frail patients is increasingly recognised as important, especially for surgery such as major elective surgery and orthopaedic trauma.

In this regard, Bagshaw and colleagues [1] present a secondary analysis of a well-conducted prospective cohort study from six intensive care units (ICUs) in Alberta, Canada during 2010 and 2011. By re-analysing data from a subgroup of 197 patients aged 50–65 years, the authors highlight novel insights relating to younger frail critically ill patients, a hitherto under-researched group.

The study reveals that frailty is prevalent in younger critically ill patients: over one quarter of patients were classified as ‘frail’ pre-hospital admission using the Canadian Study of Health and Ageing Clinical Frailty Scale (CFS) instrument and a further 34 % were classified as ‘vulnerable’. This is higher than the estimate of frailty prevalence of 10–15 % in a similar age group in a contemporaneous general Canadian population [3]. Furthermore, younger frail patients experience a substantially worse health trajectory after ICU admission, demonstrated by higher one-year mortality, higher one-year hospital readmission rates and substantially worse impairment in health-related quality of life at 6 months post-discharge.

This study contributes to the growing evidence base that ICU survivorship places a substantial burden on families and carers, including impaired physical function, neuropsychological sequelae, increased health care costs and reduced health-related quality of life [4, 5]. In addition, published guidelines have been developed advocating rehabilitation after critical illness despite conflicting results on the benefits of rehabilitation in this population [6]. Whilst a UK-based study utilising self-help manuals demonstrated positive effects on physical and psychological recovery in ICU survivors [7], studies investigating a range of post-ICU interventions demonstrate no treatment effect [8–12]. Patients post-critical illness appear to be on a trajectory of slowly improving health, which has been shown to be accelerated with exercise in one study [13] but patients appear to end up with similar levels of functioning overall. These studies demonstrate that we need a much deeper understanding of the process of recovery trajectories in physical and psychological health in this population.

The findings of this study raise important questions and have implications for clinicians and researchers in relation to recognition and management of the frail younger ICU patient. Younger frail patients form a significant cohort within our critical care units and carry an ongoing frailty burden for both themselves as well as health economies. This challenges us to examine this cohort more closely to see whether we can improve their health care trajectories post-critical illness.

Does a subgroup of the ICU population exist who may benefit from rehabilitation and if so is it the frail identified by Bagshaw and colleagues or non-frail population? Is the reason that many rehabilitation studies post-critical illness demonstrate no treatment effect because we have not yet identified those subgroups who may respond? Conceptually, the high prevalence of pre-existing frailty raises the possibility post-ICU morbidity might be attributable to the pre-admission progressive loss of physiological reserve and declining health trajectory associated with frailty rather than the acute illness. In turn, frailty may be a marker for subgroups of patients whose recovery trajectories are differentially responsive to improvement through rehabilitation interventions. In contrast, the non-frail ICU survivor group may be on a trajectory to recovery regardless of rehabilitation intervention and require nothing beyond current provision. Without additional information relating to pre-hospital admission health status of patients, it remains unclear whether the frail and non-frail groups of patients in this study were on differing trajectories before and after critical illness. Others have highlighted the risk of inaccurate inferences from outcome data after acute illness when pre-illness trajectories are inadequately measured [14].

A developing concept in perioperative medicine is that of ‘prehabilitation’ [15]. The identification of a subgroup of the population who have less physiological reserve allows clinicians to engage them in a programme of rehabilitation and exercise in the pre-surgery setting, which may improve outcome following surgery. Whilst this approach is attractive in the elective setting, it would be challenging to identify an equivalent group of younger frail individuals in the primary healthcare setting and intervene at a stage before critical illness occurs. Certainly we need to start examining this younger frail cohort in more detail to understand the reasons for their decline in physiological reserve, their health trajectories and which aspects may respond to which intervention and at what time-point, with the aim of optimising outcome and potentially decreasing utilisation of healthcare resources over the longer term, including their readmission to hospital and intensive care.
